# Book Review: Embodied Cognition (2nd Edition)

**DOI:** 10.3389/fpsyg.2020.00042

**Published:** 2020-02-19

**Authors:** Yang Yao, Xinmin Zheng

**Affiliations:** ^1^Faculty of Foreign Languages, Southwest Forestry University, Kunming, China; ^2^School of Education, Shanghai International Studies University, Shanghai, China

**Keywords:** embodied, cognition, dualism, mind-body, review (article)

Theories of embodied cognition have been around for more than 20 years and present as a superseding standard cognitive science. Without doubt, the first edition of *Embodied Cognition* published in 2011 is an unprecedented and outstanding masterpiece in this research area. In the first edition, Lawrence Shapiro sets out the central themes and debates surrounding embodied cognition, explaining and assessing the work of many of the key figures of the field.

The most conspicuous change in the second edition, however, is the greater number of chapters. The book has been updated and revised throughout; in other words, it includes new chapters that both expand on earlier topics and that introduce new material on embodied concepts, preference formation, and emotion. Helpful chapter summaries and annotated further reading have been added at the end of each chapter. The book begins with an outline of the theoretical and methodological commitments of standard cognitive science as an introduction, then examines philosophical and empirical arguments surrounding the traditional perspective, setting the stage for a detailed examination of the embodied alternative. The author also introduces topics such as dynamical systems theory, ecological psychology, robotics, and connectionism, before addressing core issues in philosophy of mind such as mental representation and extended cognition.

The body of this book is broken into 10 chapters. The first three chapters are the same as the first edition, which are standard cognitive science, challenging standard cognitive science, and concepts of embodiment. By introducing important ideas and methods within standard cognitive science through discussion of several exemplary research projects, chapters 1, 2, and 3 illustrated and examined some work that exemplifies standard cognitive science, defined what it means for cognition to be embodied, and claimed about the meaning of embodiment. The author pulls together various strands of thought and point them in the direction of embodied cognition, some ideas that might be taken to challenge the orthodox conception of cognitive science. Chapters 4 to 9 are newly modified and edited chapters. Chapters 4 and 5 explained what sense concepts may be embodied, focused on consequences that researchers take to follow from the embodiment of concepts, and claimed that it is a solution to the symbol grounding problem. Chapters 6 and 7 explicated the distinction between the format of an embodied concept and the content of the concept in the first part of the chapter, examined research that purports to show how attitudes, emotions, and moral judgments that hostage the properties of our bodies and the challenge to standard cognitive science constituted a new way of explaining or describing cognition. In chapters 8 and 9, the author further reviewed the challenge to standard cognitive science coming from the field of robotics and assessed arguments that not just the body but the world as well might be constituents of cognition. The final chapter went back to the decision tree that has been presented in the introduction, and then discussed the conceptualization, replacement, and constitution challenge to standard cognitive science as the concluding thoughts of the whole book.

Theories of embodied cognition gave psychology a new theoretical foundation and methodology; moreover, they contributed to change the basic hypotheses and the research horizons of psychology and cognitive science. Based on philosophy of mind, embodied cognitive science pushes the cognitive subject's perception and feeling of objective things to the front stage of research, and stresses the role that human body, intentionality, and subjective feeling have in the constitution of the research center. Embodied cognition theories have also shed a great significance on education. More and more researchers are interested in the interdisciplinary field of embodied cognition, and the research on the relationship between embodied cognition, education, and learning is also emerging. In the traditional classroom teaching, the body has always been considered to have nothing to do with learning, whereas embodied cognition theories combine cognition, body, and environment together. Therefore, researchers started to rethink the role of the body in education and learning with the help of embodied cognitive theory. The theory of embodied cognition is helpful for researchers to start a new exploration of teaching mode and reform the traditional teaching mode from a new perspective. Embodied cognition mainly emphasizes that cognition is formed through body structure, activity mode, and interactive experience with environment. Therefore, according to the embodied nature of education, new teaching exploration should be carried out for teachers, students, and even classroom layout, so as to achieve good teaching effect.

Nowadays, embodied cognition has become the latest trend and mainstream theory in cognitive science. There is no doubt that embodied cognition breaks through the barrier of the classical cognitive dualism of body and mind, including body and environment in cognitive processing, and promotes the development of cognitive science. However, in the process of its development, it has exposed a variety of disadvantages. How to overcome the difficulties successfully and realize the benign and sustainable development has become its primary task. Last but not least, although the environment emphasized by embodied cognition refers to both natural environment and social environment, social, and cultural factors have not been paid enough attention in embodied research. Focusing on the social, cultural, and linguistic environment and their impact on cognition could greatly implement the embodied cognition research program. Indeed, recent developments in this field are starting to deepen aspects related to these factors.

## Author Contributions

YY has written the book review. XZ is YY's supervisor, he helped to examine and modify the review.

### Conflict of Interest

The authors declare that the research was conducted in the absence of any commercial or financial relationships that could be construed as a potential conflict of interest.

